# Chemoinformatics View on Bitter Taste Receptor Agonists in Food

**DOI:** 10.1021/acs.jafc.1c05057

**Published:** 2021-11-11

**Authors:** Sebastian Bayer, Ariane Isabell Mayer, Gigliola Borgonovo, Gabriella Morini, Antonella Di Pizio, Angela Bassoli

**Affiliations:** † Leibniz Institute for Food Systems Biology at the Technical University of Munich, Lise-Meitner Str. 34, D-85354 Freising, Germany; ‡ Faculty of Life Sciences, University of Vienna, Djerassiplatz 1, 1030 Vienna, Austria; § Department of Food, Environmental and Nutritional Sciences-DeFENS, University of Milan, via Celoria 2, 20147 Milano, Italy; ∥ University of Gastronomic Sciences, piazza Vittorio Emanuele 9, 12042 Pollenzo, (Bra, CN), Italy

**Keywords:** bitter molecules, food, bitter taste receptors, TAS2Rs, scaffold decomposition, chemical space

## Abstract

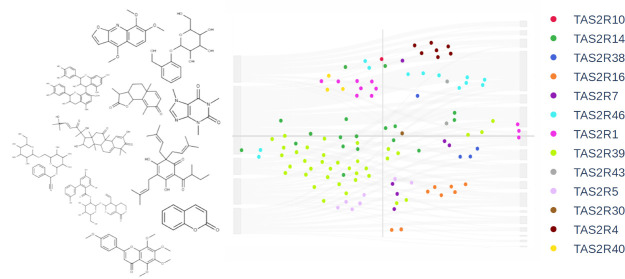

Food compounds with a bitter taste have a role in human health, both for their capability to influence food choice and preferences and for their possible systemic effect due to the modulation of extra-oral bitter taste receptors (TAS2Rs). Investigating the interaction of bitter food compounds with TAS2Rs is a key step to unravel their complex effects on health and to pave the way to rationally design new additives for food formulation or drugs. Here, we propose a collection of food bitter compounds, for which in vitro activity data against TAS2Rs are available. The patterns of TAS2R subtype-specific agonists were analyzed using scaffold decomposition and chemical space analysis, providing a detailed characterization of the associations between food bitter tastants and TAS2Rs.

## Introduction

In humans, bitter taste is mediated by 25 bitter taste receptors (TAS2Rs), belonging to the superfamily of G protein-coupled receptors.^1−3^ Bitter taste is generally considered as an aversive reaction that protects humans from ingesting toxic compounds. However, neither all compounds that are bitter are toxic nor do all toxins taste bitter.^4^ Many bitter compounds, such as polyphenols, glucosinolates, and terpenes, have proven beneficial health effects,^5^ and diets including higher amounts of bitter-tasting foods have been associated with better health.^6,7^

Babies have an innate preference for sweet, savory, and fatty tastes, while they reject even low levels of bitterness (and acidity). In fact, liking bitter-tasting foods is a learned behavior, and studies demonstrated that learning plays a major role in what comes to be identified as “food” and how the taste education in babies (especially improving bitter taste acceptance) could have a role on the health of the future adults.^8−10^

Interestingly, bitterness intensity is reported to have a special role in the food-medicine continuum: mild bitterness is associated with plants used as food, medium bitter taxa are seen both as food and medicine, while plants perceived to be very bitter are considered to be only medicinal.^11^ This traditional knowledge and wisdom found a possible explanation on the fact that TAS2Rs have been discovered in several extra-oral tissues, including the gastrointestinal (GI), respiratory, reproduction, and urinary systems.^12−16^ In these extra-oral locations, they may interact with endogenous compounds (produced by the microbiota and pathogens) and exogenous ones (such as bitter compounds in food), in both cases mediating systemic response, ranging from innate immunity^13,17^ to metabolic effects.^18−21^ GI bitter taste receptors are supposed to play a role (or at least to contribute) in maintaining a salutary balance among a healthy microbiome, diet, and weight.^22^ Due to these recent discoveries, bitter taste receptors become interesting targets to improve human health, both through the diet (development of functional foods)^23−25^ and as new drugs targets.^20,26,27^

Investigating the interaction between bitter food compounds and TAS2Rs is a key step to decode their complex effects on health and to pave the way to rationally design new additives for food formulation. More than 1000 compounds are reported to taste bitter.^28^ Some of them are known for a long time when the practice of tasting pure compounds from isolation or synthesis was a quite common procedure. For instance, the taste of purified compounds—as recorded by the author—is usually described in the Beilstein Handbuch der Organischen Chemie (Handbook of Organic Chemistry), one of the oldest collections of organic compounds information edited from 1880 to 1998.^29^ The compounds of particular interest in the food industry have been re-tested afterward using current sensory analysis methods.^30^ Following the discovery of TAS2Rs, the associations with the cognate receptor(s) were investigated for many bitter compounds, including drugs—either from natural or synthetic origin—and food components. In vitro assays can be run on milligram scale samples and do not require preliminary toxicity tests.^31^ Some of the recently identified TAS2R agonists have not even been submitted to sensory analysis.^32,33^ Functional assays led to a new array of data connected to bitter compounds, and for a better understanding of the molecular recognition of bitter compounds, supporting the combinatorial coding of the bitter taste percept. Some bitter receptors are broadly tuned and respond to a wide range of structurally diverse bitter compounds, whereas others are very selective for one or a small number of compounds.^34^ Interestingly, some bitter compounds can act as agonists for certain TAS2R subtypes and antagonists for others.^35^ Moreover, TAS2Rs may work as heterooligomers,^36^ and TAS2R subsets have been found to be co-expressed in the taste receptor cells of human circumvallate papillae, suggesting that cells are tuned for the subsets of bitter stimuli.^37^ Several studies have been focusing on investigating the activation of specific TAS2Rs by the individual classes of bitter compounds.^38−40^ Here, we propose a systematic chemoinformatic analysis of bitter compounds present in food for which activity data against TAS2Rs are available. The analysis of the patterns of TAS2R agonists provide a detailed characterization of associations between food bitter compounds and TAS2Rs, and a framework for chemoinformatics works on the growing number of food bitter compounds.

## Materials and Methods

A total of 247 natural compounds with TAS2R activity data were collected from the literature.^41^ Canonical SMILES (simplified molecular-input line-entry system) were retrieved from PubChem. According to their presence in food sources, a set of 138 food bitter compounds was established (available at https://github.com/dipizio/Natural_TAS2R_agonists). 133 out of 138 were successfully classified using the chemical taxonomy analysis by the ClassyFire Batch compound classification tool (https://cfb.fiehnlab.ucdavis.edu/).^42^ This set of 133 compounds was used for the following analyses.

Unique scaffolds were calculated using the Bemis–Murcko Scaffold decomposition tool available in Maestro (Schrödinger Release 2021-2: Maestro, Schrödinger, LLC, New York, NY, 2021).

MACCS fingerprints were calculated from the Canonical SMILES using RDKIT in KNIME 4.3.0. The t-distributed stochastic neighbor embedding (t-SNE) for chemical space visualization was obtained using the t-SNE (L. Jonsson) node in KNIME 4.3.0. The following parameters were set for the t-SNE analysis: dimensions to reduce: 2, iterations 5000, θ: 0.5, number of threads: 8, seed: 1615892099957. The t-SNE plot was then visualized with Plotly Express.

The alluvial plot was generated with Plotly Express. The nodes settings were as followes: thickness of 20, pad to 10, and uniform color of “gray”. TAS2Rs targeted were set as the primary source (left nodes), ClassyFire superclasses of the compounds as the primary target/secondary source (middle nodes), and the number of receptors targeted per compound was set as the secondary target (left nodes). TAS2Rs were sorted using promiscuity indices (TAS2R14, −1, −46, −4, −39, −10, −5, −7, −40, −43, −16, −38, −30, −8, −31, −20, −50), superclasses by the number of compounds in each group, from largest to smallest, and compound promiscuity in increasing order (compounds that activate only one receptor, and compounds that activate two, three, four, or more than five receptors).

The following Python packages were used: NumPy, pandas, Matplotlib, Plotly Express, and Seaborn.

## Results and Discussion

### TAS2R Agonists in Food

An extensive literature search led us to collect 247 bitter compounds from natural sources from which receptor activation data are available (https://github.com/dipizio/Natural_TAS2R_agonists). To the best of our knowledge, this is the largest collection of bitter natural compounds with associated TAS2Rs’ activity values. Currently, the BitterDB has 58 compounds labeled as natural compounds (http://bitterdb.agri.huji.ac.il/, as of August 2021).^43^ Of the 25 human TAS2Rs, 21 have been de-orphanized over the last two decades with both natural and synthetic compounds.^44,45^ As reported by a number of chemical space studies, synthetic and natural compounds may chemically differ.^46−48^ Therefore, we aim to focus our analyses on the bitter molecules of natural sources. Most of the collected bitter compounds are from plant origin, with the exception of a few amino acids and peptides that can be found both in plants and in animal products (like cheese). More specifically, our analyses focused on bitter molecules in food. The definition of “food” itself can be troublesome: in fact, some plants can be regarded as food only in case of lacking other sources (alimurgic plants),^49^ some are used both as food and as medicines according to their ecosystem and culture.^50^ In our classification, we tried to focus on bitter compounds in food commonly used in the western diet. A subset of 133 food bitter compounds with receptor information (food TAS2R agonists’ dataset, available at https://github.com/dipizio/Natural_TAS2R_agonists) was built. The compounds of this set activate 17 bitter taste receptors.

The number of food TAS2R agonists associated with the receptor subtypes is reported in Table 1. For each set of TAS2R agonists, we calculated the number of unique scaffolds. Scaffold decomposition focuses on the ring systems (scaffolds), and for this reason, it is used as a measure of the chemical complexity.^51^ The ratio of the number of scaffolds by the number of compounds gives rise to the “scaffold per compound”: receptors with values higher than one are activated by chemically complex compounds (more scaffolds than compounds). The receptor with the highest “scaffold per compound” value is the TAS2R50. The high number of scaffolds is attributed to the size of amarogentin (MW of 586), the only TAS2R50 agonist in the food TAS2R agonists’ dataset. Amarogentin is considered the most bitter natural molecule known to date^52,53^ and targets eight TAS2Rs in total (https://github.com/dipizio/Natural_TAS2R_agonists). A high value of “scaffold per compound” is assigned also to TAS2R5, which is activated by complex polyphenolic structures, with a median MW of 578. Instead, in the case of TAS2R43, the “scaffold per compound” higher than 1 is associated with a rather low molecular size (median MW of 261), indicating a chemical diversity of the set of agonists. Among all receptors, TAS2R1, TAS2R14, and TAS2R16 have a “scaffold per compound” value lower than 1 (more compounds than scaffolds), pinpointing a chemical similarity of their agonists.

**Table 1:** TAS2Rs Activated by the Food Bitter Compounds (All-TAS2R-Tested Agonists)

receptor (alternative names)	n. of compounds	n. of unique scaffolds	scaffold per compound	median MW
TAS2R1	25	20	0,80	354
TAS2R4	14	34	2,43	586
TAS2R5	9	52	5,78	578
TAS2R7	10	26	2,60	535
TAS2R8	2	2	1,00	160
TAS2R10	12	20	1,67	410
TAS2R14	78	53	0,68	295
TAS2R16	8	6	0,75	318
TAS2R20 (TAS2R49, TAS2R56)	1	1	1,00	204
TAS2R30 (TAS2R47)	4	14	3,50	477
TAS2R31 (TAS2R44, TAS2R53)	2	3	1,50	271
TAS2R38 (TAS2R61)	5	6	1,20	163
TAS2R39 (TAS2R57)	47	66	1,40	286
TAS2R40 (TAS2R58)	8	8	1,00	354
TAS2R43 (TAS2R52)	8	19	2,38	261
TAS2R46 (TAS2R54)	20	31	1,55	335
TAS2R50 (TAS2R51)	1	9	9,00	586

### Broad- and Fine-Tuning of TAS2Rs Versus Food Bitter Compounds

**Figure 1:**
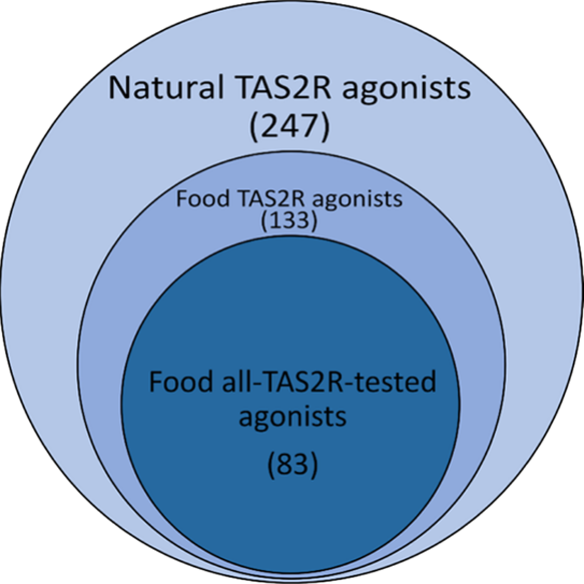
Venn diagram of the three datasets collected and analyzed in this work: TAS2R agonists from natural sources (in light blue), TAS2R agonists from food sources (in blue), and TAS2R agonists from food sources that were tested toward all bitter taste receptors (in dark blue). Datasets are available at https://github.com/dipizio/Natural_TAS2R_agonists

**Figure 2:**
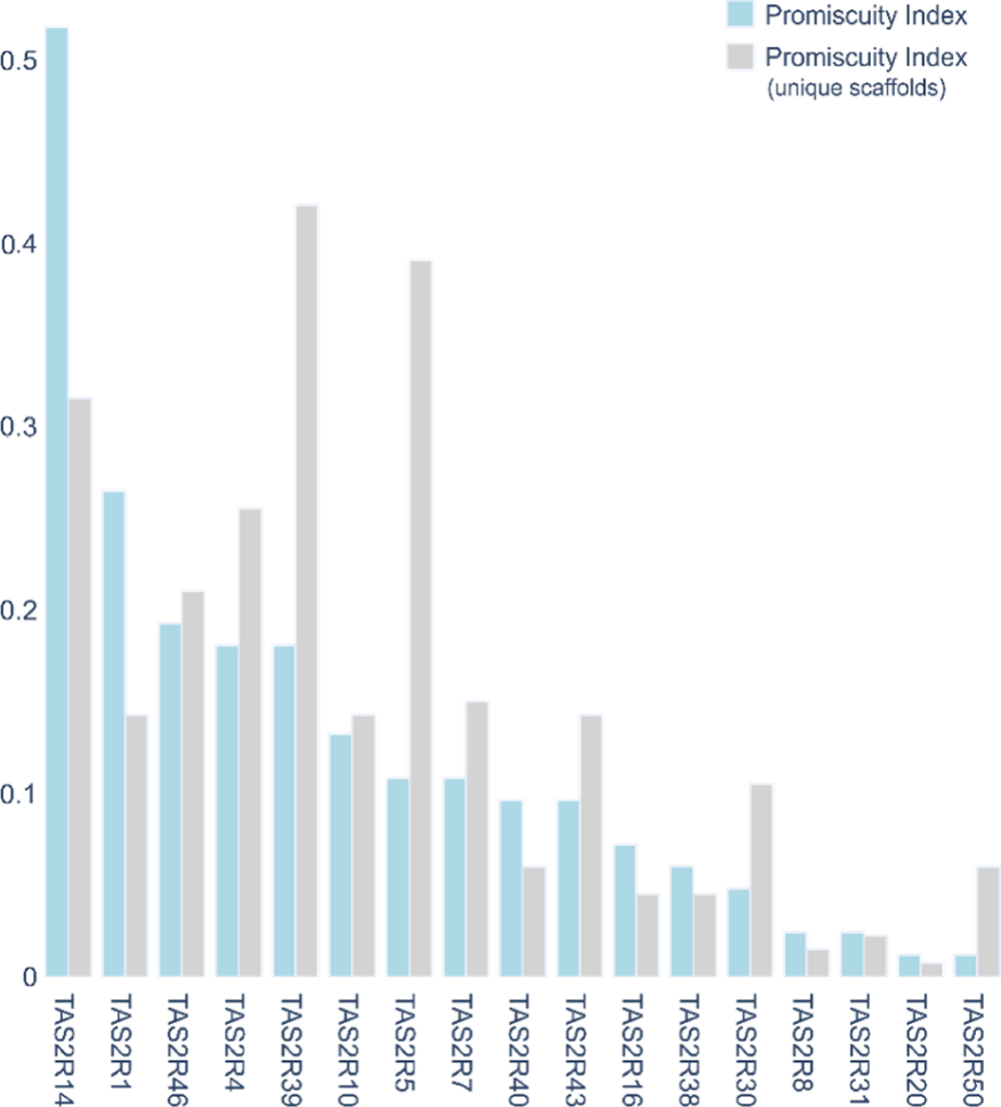
Receptor promiscuity indexes of bitter taste receptors toward the food all-TAS2R-tested agonists’ set. Light blue bars indicate the number of compounds that activate the individual TAS2R divided by the total number of molecules; while gray bars represent the number of unique scaffolds for each receptor divided by the total number of unique scaffolds.

Whereas the scaffold decomposition analysis of the entire dataset was aimed to investigate the TAS2R agonist sets, to compare the agonists of individual receptors, the exclusion of molecules tested only against certain subtypes was needed. Therefore, a smaller subset of 83 compounds tested toward all TAS2Rs was extracted (activity value range: 1–10 000 μM): we will refer to this subset as the “food all-TAS2R-tested agonists’ dataset”. Figure 1 shows the three sets of compounds used for the different analyses. To compare the receptive ranges of TAS2Rs, we defined the promiscuity index (light blue bars in Figure 2) as the number of bitter compounds that activate the individual TAS2R divided by the total number of molecules (food all-TAS2R-tested agonists’ dataset).^34^ To represent the diversity of the ligands set, we also calculated the number of unique scaffolds for each receptor divided by the total number of unique scaffolds, that is, 133 (gray bars in Figure 2). The height of the bars depends on the number of compounds and scaffolds of the individual receptors but also on the total numbers of molecules (i.e., 83) and scaffolds (i.e., 133); therefore, promiscuity indices per number of compounds and per scaffolds are not directly comparable. However, with this analysis we want to focus on the comparison of the different TAS2R subtypes and, therefore, on the profiles of the histograms we obtain from the analyses of the number of compounds and number of scaffolds.

TAS2R14, activated by almost half of the food all-TAS2R-tested agonists’ dataset, results to be the most broadly tuned receptor. TAS2R14 is the most promiscuous receptor also when synthetic compounds are taken into account.^34^ TAS2R1 follows with about half as many agonists as TAS2R14. Intermediate-promiscuous TAS2R receptors, that is, TAS2R46, TAS2R4, and TAS2R39, follow with a promiscuity index of about 0.2. The most selective receptors are TAS2R50 and TAS2R20. Interestingly, the profile is different when looking at scaffolds. TAS2R39 and TAS2R5 are more enriched in scaffolds compared to TAS2R14. As already discussed, the high number of unique scaffolds of TAS2R5 is due to the complexity of its ligands. On the contrary, the promiscuity index calculated for the unique scaffolds of TAS2R39 agonists is higher than that of TAS2R14 agonists, although the median MW of TAS2R14 and TAS2R39 ligands is comparable.

### Chemical Classes Associated with TAS2R Subtypes

The chemical classes associated with TAS2R agonists in food were assigned using the superclasses of ClassyFire, as previously done for chemical classification analyses of bitter compounds.^53,54^ The ClassyFire provides an automated chemical classification based on a structure-based chemical taxonomy consisting of approximately 5000 different categories.^42^ The ClassyFire chemical taxonomy consists of up to 11 different levels (kingdom, superclass, class, subclass, etc.). In our case, we decided to use the superclasses, in order to have a meaningful but simple grouping of our datasets.

The food TAS2R agonists’ dataset is composed of phenylpropanoids and polyketides (with 61 compounds this superclass makes up about half of the whole set), lipids and lipid-like molecules (31), organic oxygen compounds (12), organic acids (10), organoheterocyclic compounds (8), benzenoids (8), mixed metal/nonmetal compounds (2), organosulfur compounds (1, i.e., allyl isothiocyanate), and alkaloids (1, i.e., quinine). The food all-TAS2R-tested agonists’ dataset has a substantial reduction of phenylpropanoids and polyketides to about a fourth (22), but the distribution of other superclasses is rather similar: lipids and lipid-like molecules (33), organic oxygen compounds (10), benzenoids (7), organic acids (7), organoheterocycles (5), mixed metal/nonmetal compounds (2), organosulfur compounds (1), and alkaloids (1).

**Figure 3:**
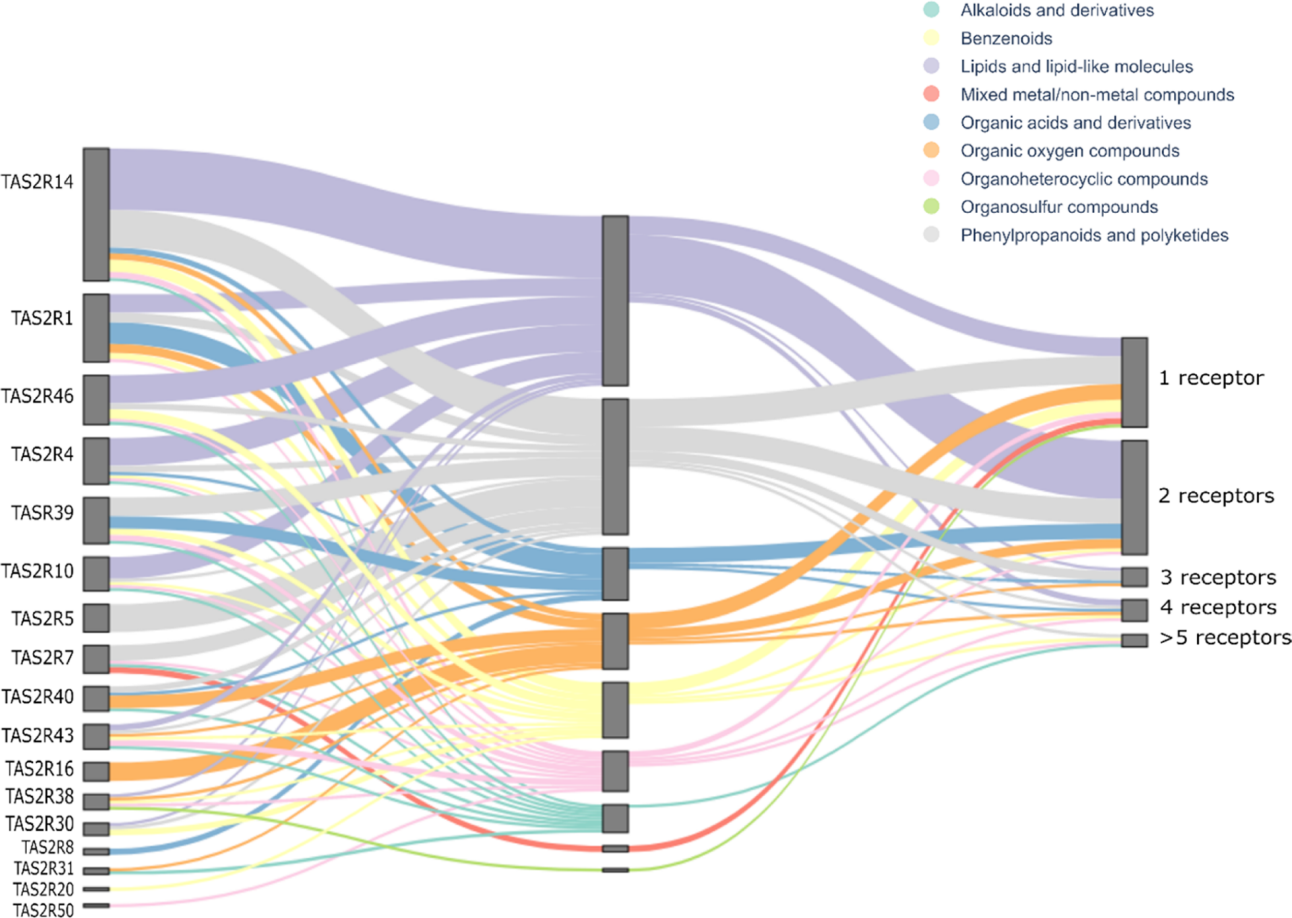
Alluvial plot depicting the associations of food bitter compounds with bitter taste receptors. On the left, receptors are ordered by their promiscuity (i.e., the number of compounds that activate each TAS2R); on the right, compounds (food all-TAS2R-tested agonists’ set) are ordered by their promiscuity (i.e., how many receptors each compound can activate). The different colors of flows represent the superclasses (central nodes of the plot) the compounds are associated with.

To investigate the target promiscuity of TAS2Rs toward chemical superclasses, we analyzed the associations of food all-TAS2R-tested agonists’ with the receptors (Figure 3). The outstanding promiscuity of TAS2R14 reflects here again, as it is the only channel that has ligands from seven superclasses. TAS2R1, TAS2R4, and TAS2R43 follow with agonists from six superclasses. In the middle, with compounds from four and five superclasses, are TAS2R46, TAS2R39, TAS2R10, TAS2R38, TAS2R7, and TAS2R40. TASR38 stands out as it only has five agonists in our set, each of them belonging to a different superclass. Among the very selective receptors, TAS2R8 and TAS2R31 are activated by two compounds, while TAS2R20 and TAS2R50 by a single agonist, l-tryptophan^55^ and amarogentin,^56^ respectively, which target multiple other TAS2Rs. Interestingly, we have two receptors activated by one single superclass of compounds, TAS2R16 and TAS2R5. TAS2R16 is activated by six different compounds, all organic oxygen compounds. The similarity of TAS2R16 agonists was highlighted also with the scaffolds analysis, and it is well renowned because TAS2R16 is specialized in the recognition of “bitter sugars”,^57^ as glucopyranosides^38^ and glucosinolates.^32^ Similarly, TAS2R5 shows specificity toward polyphenols: it is activated only by compounds belonging to the phenylpropanoids and polyketides superclass. This indication could be relevant for TAS2R5-targeted nutritional studies in the future because polyphenols are biomolecules with a well-established positive impact on nutrition and health.^58−60^ Moreover, TAS2R5 is expressed in the small and large intestine and it may be a player in recognizing bitter polyphenols in the diet with positive effects on health.^61^

The right side of the alluvial plot in Figure 3 reports the compound promiscuity/selectivity, that is, the number of receptors each compound in the food all-TAS2R-tested agonists’ set can activate. Interestingly, as observed analyzing also synthetic compounds,^34^ promiscuous compounds can activate both promiscuous and selective receptors. Most of the compounds activate one or two receptors, and compound promiscuity is not driven by specific superclasses. Superclasses are distributed over all five promiscuity groups, and very selective compounds (those activating only one receptor) belong to all superclasses. Superclasses are defined in the middle nodes of the plot (Figure 3). The mixed metal/nonmetal (i.e., CaCl_2_ and MgCl_2_) and the organosulfur (i.e., isothiocyanate) superclasses show the highest specificity of the superclasses, targeting only TAS2R7^62,63^ and TAS2R38,^39,64^ respectively. The phenylpropanoids are at the other end of the spectrum activating a total of 11 receptors, followed by the organoheterocyclic compounds and benzenoids with 10 associated TAS2Rs. Most of the superclasses activate between six and nine receptors. Interestingly, the number of molecules in a given superclass is not the only driver behind compound promiscuity, for example, 44 compounds belong to the lipids and lipid-like superclass and activate eight receptors, whereas quinine, the single compound classified as alkaloid, activates nine receptors.

### Chemical Space of Bitter Compounds in Food

**Figure 4:**
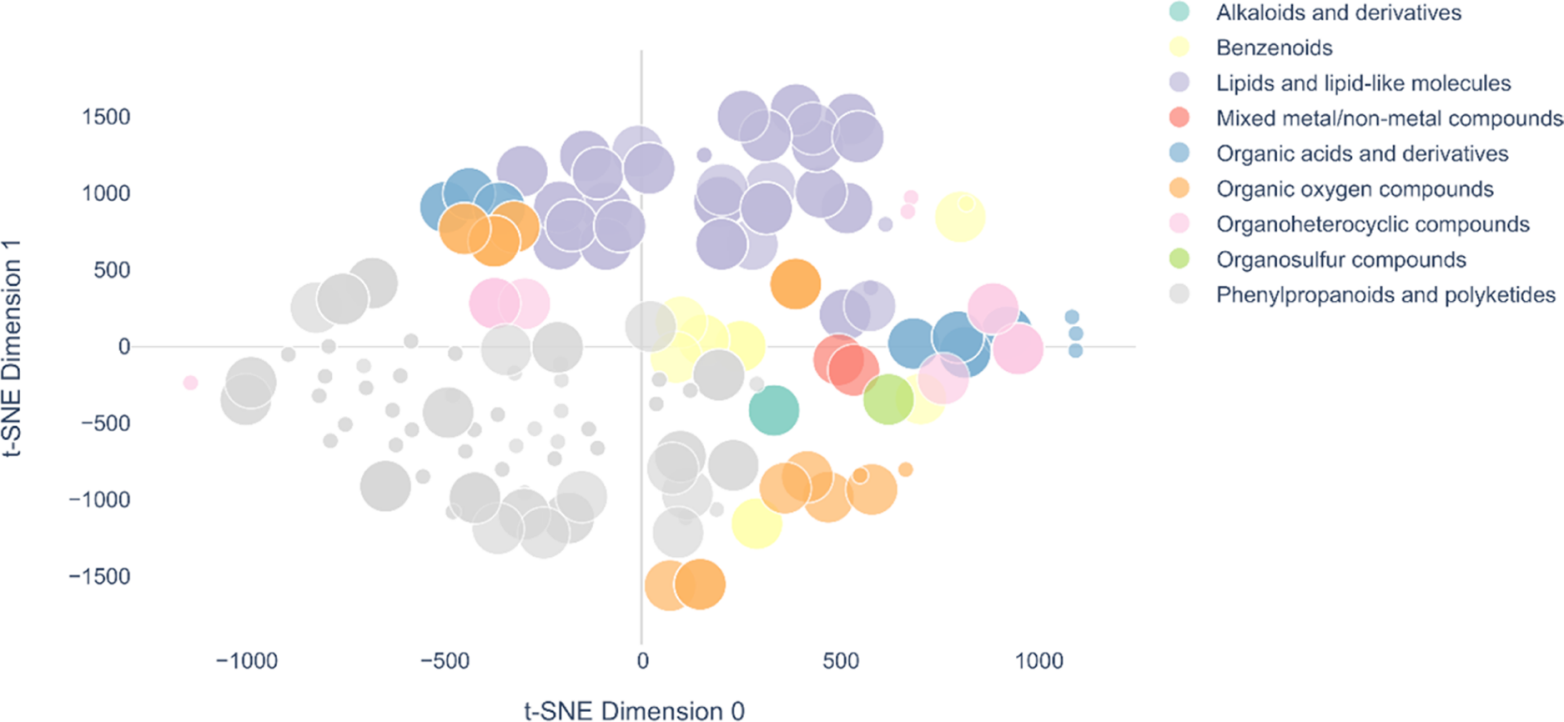
t-SNE plot of the food all-TAS2R-tested agonists. Compounds are colored according to the ClassyFire superclasses and sized in a bigger size when belonging to the food all-TAS2R agonists’ set. t-SNE dimension 0 and 1 for all compounds reported in this plot are available at https://github.com/dipizio/Natural_TAS2R_agonists.

The classification into superclasses, which includes 26 organic and 5 inorganic generic categories,^42^ necessarily leads to groups that do not completely reflect a rationale in the chemistry of compounds: for instance, only quinine is classified as alkaloid, whereas caffeine is among heterocycles; allyl isothiocyanate is in sulfur compounds, whereas phenethyl isothiocyanate is within benzenoids, and the glucosinolates sinigrin and glucoputranjivin, which also contain a sulfur atom, are in the group of oxygenated compounds. In order to better understand the chemical similarity within and between superclasses, we analyzed the chemical space of the food TAS2R agonists’ set (Figure 4). Specifically, a fingerprint-based chemical space was built and visualized by t-SNE, a machine learning algorithm for data visualization developed by van der Maaten and Hinton in 2008.^65^ The t-SNE performs a nonlinear projection of data points from a high-dimensional space to a low-dimensional space. Our main goal for building a chemical space was to investigate the chemical similarity of compounds in our dataset and attempt to identify (chemistry-driven and/or receptor-driven) subgroups within it. Compared to other dimensionality reduction methods, such as the principal component analysis, that concentrate on placing dissimilar data points far apart, the t-SNE tends to place similar data points close together, and we found it best fitting our aim.

In the t-SNE plot in Figure 4, the compounds are colored according to the superclasses and sized according to their belonging to the food all-TAS2R-tested (big dots) or food TAS2R (small dots) agonists. Interestingly, the food all-TAS2R-tested agonists’ set has similar distribution in the chemical space as the food TAS2R agonists’ set and a similar representation of superclasses. Indeed, most often the experimental screening of certain compounds toward specific TAS2Rs is focused on chemotypes that were previously screened toward all receptors.^40,66^ Interestingly, with some exceptions, molecules within the same superclasses map the same regions of the chemical space. The largest superclass, phenylpropanoids and polyketides (in gray in Figure 4) span all around the lower left-hand side of the plot, while lipids and lipid-like molecules (in violet) spread on the positive side of the t-SNE-1 axis. The two mixed metal/nonmetal compounds (red dots) cluster in the right-center, very close to each other. Benzenoids (yellow dots) spread on the right-hand side of the plot, the core cluster in the center is mapped by one-ring structures (i.e., phenylethyl isothiocyanate, protocatechuic acid, pyrocatechin, salicylic acid, and vanillic acid), while the more complex structures of amarogentin, 11β,13-dihydrolactucopicrin, and lactucopicrin are the dots spreading the bottom and left side, respectively. The organosulfur compound allyl isothiocyanate (green) is very close to the structurally related phenylethyl isothiocyanate (yellow dot), which is indeed isolated from the other benzenoids. The t-SNE analysis, therefore, correctly associates compounds formally assigned to different superclasses but with the same functional groups. The organoheterocyclic compounds (eight pink dots in the plot) spread over the plot. This was expected because the heterocycle superclass includes very different oxygenated, nitrogen and other five- and six-member ring compounds. The pink dots form distinct groups: caffeine close to theobromine, lactucin close to 11β,13-dihydrolactucin, ethylpyrazine close to thiamine and l-tryptophan, and skimmianine, an alkaloid isolated from *Ruta graveolens* L., a plant having occasional uses as a food,^33^ isolated on the extreme left side of the plot.

Likewise, structurally similar compounds belonging to different superclasses are associated by vicinity in the t-SNE plot. The hop-derived humulones (humulone, adhumulone, and cohumulone) are labeled as organic oxygen compounds and are in the upper left corner of the t-SNE plot (orange dots), directly opposite to three lupulones (adlupulone, colupulone, and lupulone), labeled as organic acids (blue dots). Lupulones are indeed structurally very similar to humulones and less similar to other organic acids, amino acids, and di/tripeptides that are placed on the right-hand side of the chemical space.

**Figure 5:**
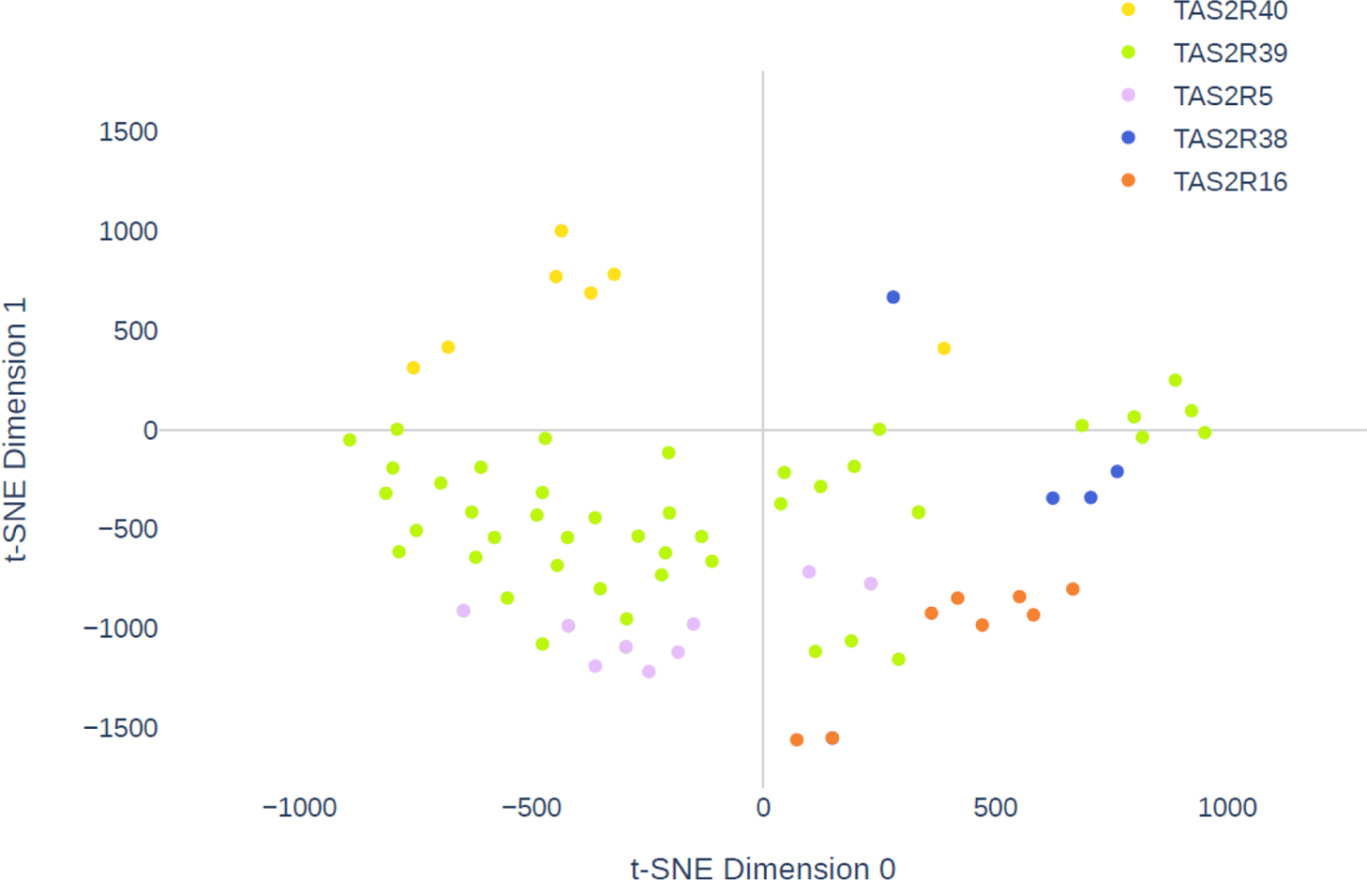
Receptor space of TAS2R40, TAS2R39, TAS2R5, TAS2R38, and TAS2R16 agonists.

Coloring compounds in the t-SNE plot according to cognate receptors provides a view of the “receptor space” covered by food bitter compounds. In Figure 5, only TAS2R39, TAS2R5, TAS2R16, TAS2R38, and TAS2R40 ligands are plotted, for a clearer definition of the formed groups, but all individual agonists are reported in the Supporting Information Figures S1–S17. TAS2R14 agonists are the most numerous and occupy a large portion of the plot. TAS2R1, TAS2R4, TAS2R10, and TAS2R46 agonists come from many different superclasses (Figure 3) and are spread over the plot. TAS2R39 ligands mostly occupy the region of phenylpropanoids and polyketides in Figure 4. In the same area, at the bottom, TAS2R5 agonists cluster together (lilac dots). TAS2R16 agonists, all organic oxygen compounds, are also very close in space (orange dots); only two molecules, sinigrin and glucoputranjivin, are distant from the main group, reasonably because they both contain a sulfurated group. TAS2R40 ligands cluster together at the left side of the plot (yellow dots), with the exception of quinine and pantothenic acid. Three of the five TAS2R38 agonists group together closely, that is, allyl isothiocyanate, phenylethyl isothiocyanate, and ethylpyrazine, whereas limonin and sinigrin are structurally very different and distant from the core cluster (see Figure S9).

**Figure 6:**
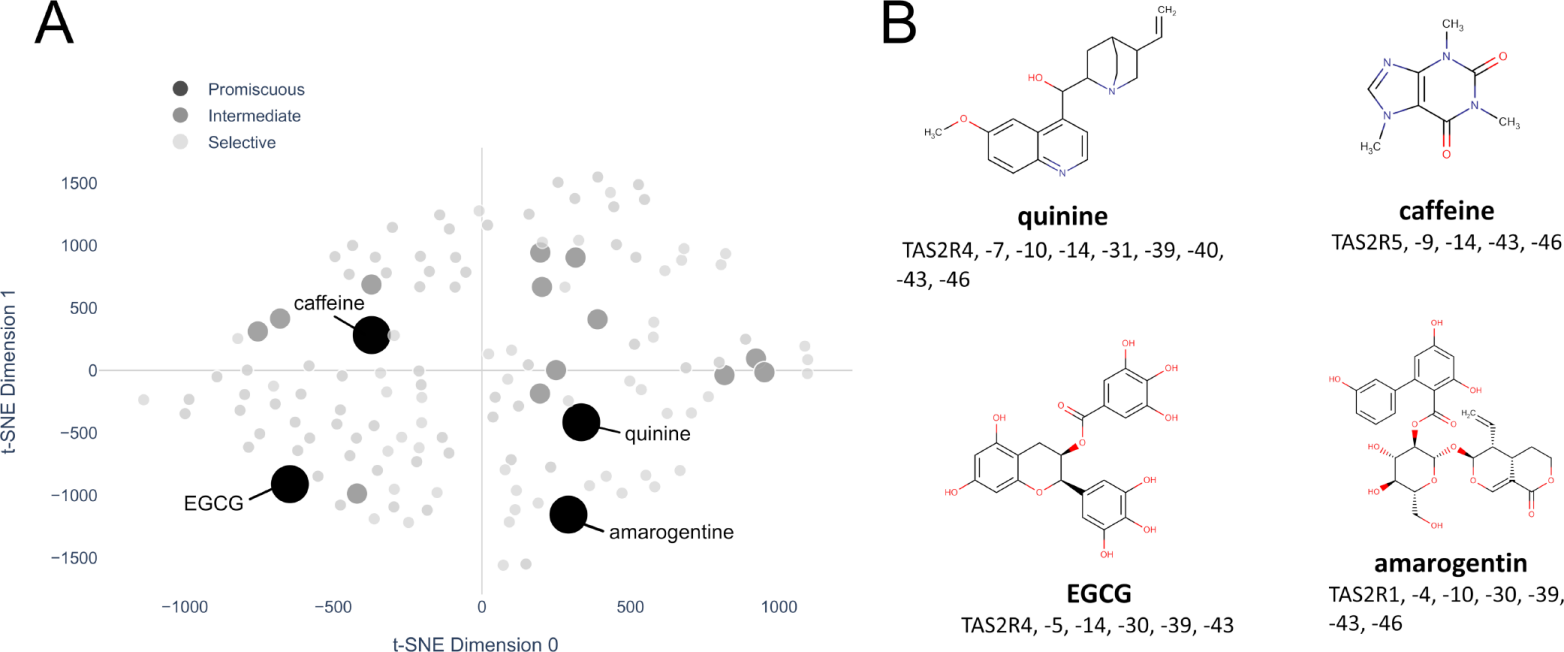
(A) t-SNE plot with compounds colored and sized according to their promiscuity: promiscuous compounds (activating more than five receptors) in black, intermediate (three and four receptors), and selective bitter compounds (one and two receptors). (B) Chemical structures of the most promiscuous compounds, that is, quinine, amarogentin, EGCG, and caffeine.

As previously shown in Figure 3, the promiscuity and selectivity of bitter compounds cannot be linked by superclasses. Indeed, annotating compounds in the t-SNE plot by their promiscuity, we do not recognize promiscuous-specific or selective-specific regions, but all types of compounds spread in the chemical space (Figure 6A). On the other hand, very similar compounds may have different promiscuity profiles: caffeine and theobromine are structurally very similar, are in the same superclass and very close in space in the t-SNE plot, but one is selective for a single receptor and the other is very promiscuous.

The four most promiscuous compounds are quinine, amarogentin, epigallocatechin gallate (EGCG), and caffeine (Figure 6). Quinine activates nine receptors, that is, TAS2R4, TAS2R7, TAS2R10, TAS2R14, TAS2R31, TAS2R39, TAS2R40, TAS2R43, and TAS2R46, amarogentin seven receptors, that is, TAS2R1, TAS2R4, TAS2R10, TAS2R30, TAS2R39, TAS2R43, and TAS2R46, EGCG six receptors, that is, TAS2R4, TAS2R5, TAS2R14, TAS2R30, TAS2R39, and TAS2R43, and caffeine five receptors, that is, TAS2R5, TAS2R9, TAS2R14, TAS2R43, and TAS2R46. To be noted as TAS2R43 is a receptor shared by all four promiscuous compounds. The low number of receptor data does not allow us to drive conclusions about the roles of specific receptors for the recognition of food bitter compounds, and indeed more information is needed to see if our observations can be turned into assumptions.

In conclusion, the collection of data about TAS2R agonists in food and their analyses provide a chemical re-organization of current data and a clearer picture of the associations between food bitter compounds and their receptors. The number of compounds analyzed in this paper (133) is still quite low, but a very much higher number of TAS2R agonists is expected to be identified in food as far as the isolation of single compounds and in vitro assays on TAS2Rs will be performed. This will allow further studies and refinements on the structural classification of bitterants in relationship with their receptors. In the past, bitter compounds were identified mainly using sensory methods, which likely detect compounds with the lower recognition threshold. Further investigations aimed at identifying compounds with a mild bitter taste, difficult to be identified using sensory and taste-guided analysis, could deepen the knowledge of TAS2R agonists in food and may represent an opportunity to detect biomolecules with potential beneficial effects on health, and therefore applications in food science, nutrition, and medicine.

## Supporting Information Available

TAS2R14 agonists’ space; TAS2R39 agonists’ space; TAS2R5 agonists’ space; TAS2R16 agonists’ space; TAS2R46 agonists’ space; TAS2R4 agonists’ space; TAS2R10 agonists’ space; TAS2R1 agonists’ space; TAS2R38 agonists’ space; TAS2R30 agonists’ space; TAS2R40 agonists’ space; TAS2R43 agonists’ space; TAS2R7 agonists’ space; TAS2R50 agonist’s space; TAS2R31 agonists’ space; TAS2R31 agonists’ space; TAS2R20 agonist’s space (PDF)
The database of natural TAS2R agonists and datasets used for the analyses are available at https://github.com/dipizio/Natural_TAS2R_agonists
