# Supplementary material for: Chemoinformatics View on Bitter Taste Receptor Agonists in Food
Source: J Agric Food Chem. 2021 Nov 11;69(46):13916–24. doi: 10.1021/acs.jafc.1c05057 (PMC8630789; doi:10.1021/acs.jafc.1c05057)

Supporting Information

A Chemoinformatics View on Bitter Taste Receptor Agonists in Food

Sebastian Bayer,[1,2,$] Ariane Isabell Mayer,[3,$] Gigliola Borgonovo,[3] Gabriella Morini,[4] Antonella Di Pizio,[1,*] Angela Bassoli[3,*]

[1]Leibniz-Institute for Food Systems Biology at the Technical University of Munich, Lise-Meitner Str. 34, D-85354 Freising, Germany

[2]University of Vienna, Faculty of Life Sciences, Djerassiplatz 1, 1030 Vienna

[3]University of Milan, Department of Food, Environmental and Nutritional Sciences-DeFENS, via Celoria 2, 20147 Milano, Italy

[4]University of Gastronomic Sciences, piazza Vittorio Emanuele 9, 12042 Pollenzo, (Bra, CN) Italy

[$] These authors contributed equally to this work.

* corresponding authors; E-MAIL: a.dipizio.leibniz-lsb@tum.de; angela.bassoli@unimi.it

Figure S1: TAS2R14 agonists' space

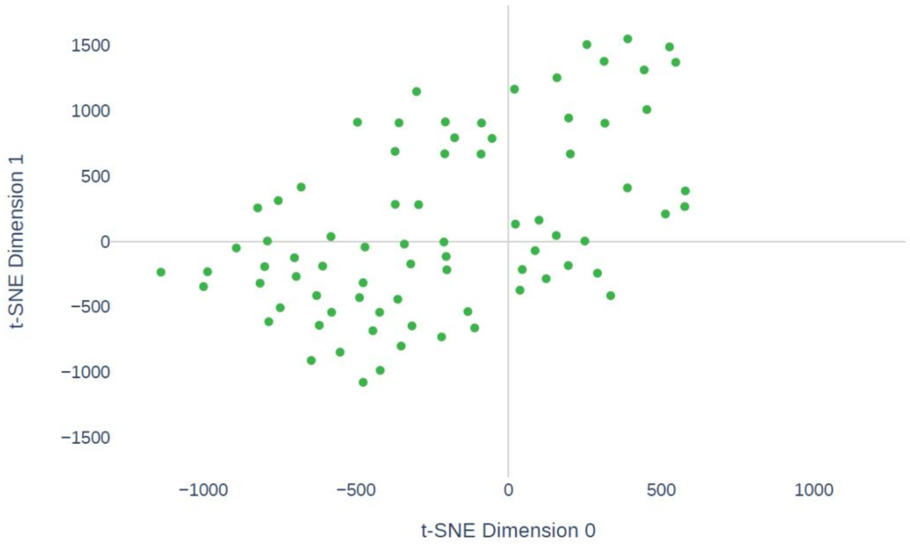

Figure S2: TAS2R39 agonists' space

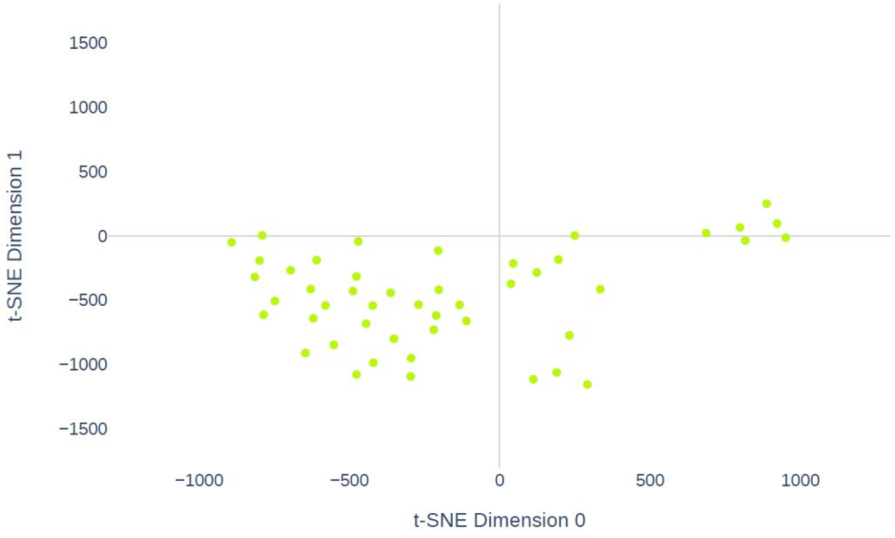

Figure S3: TAS2R5 agonists' space

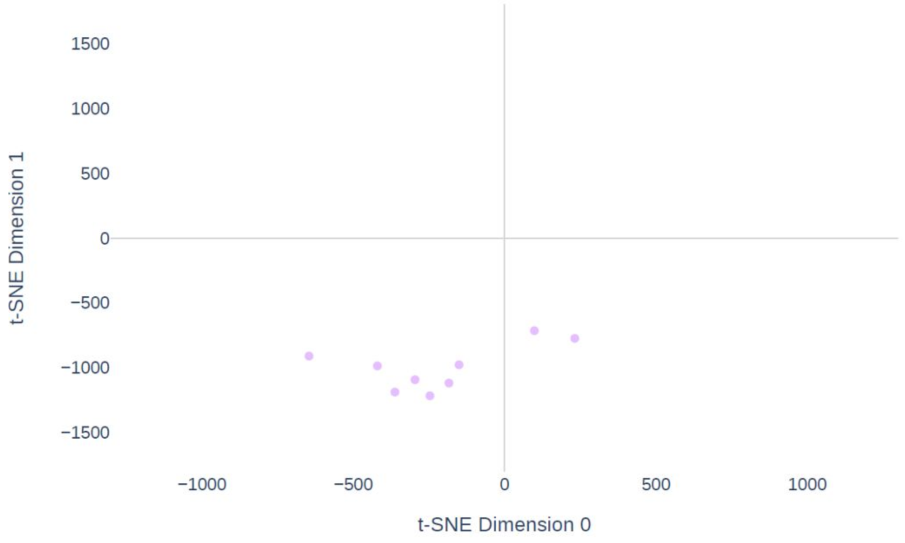

Figure S4: TAS2R16 agonists' space

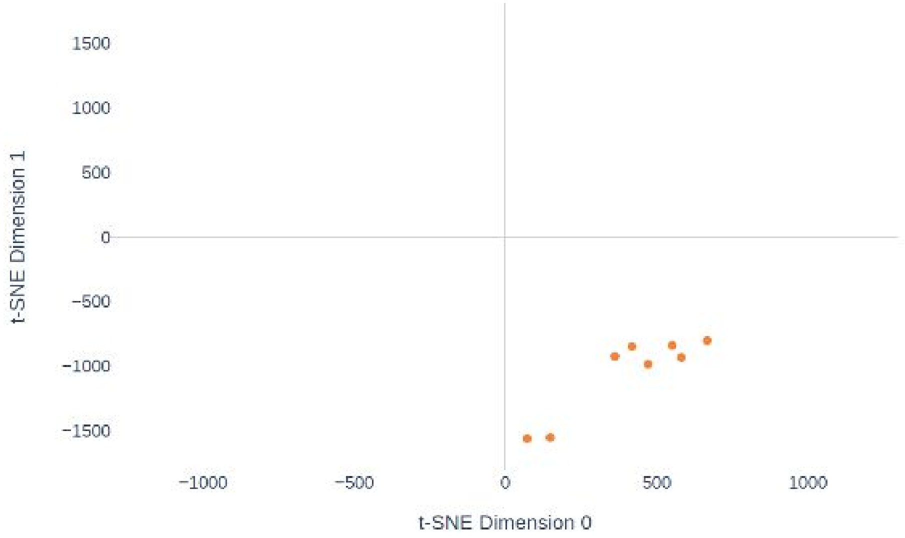

Figure S5: TAS2R46 agonists' space

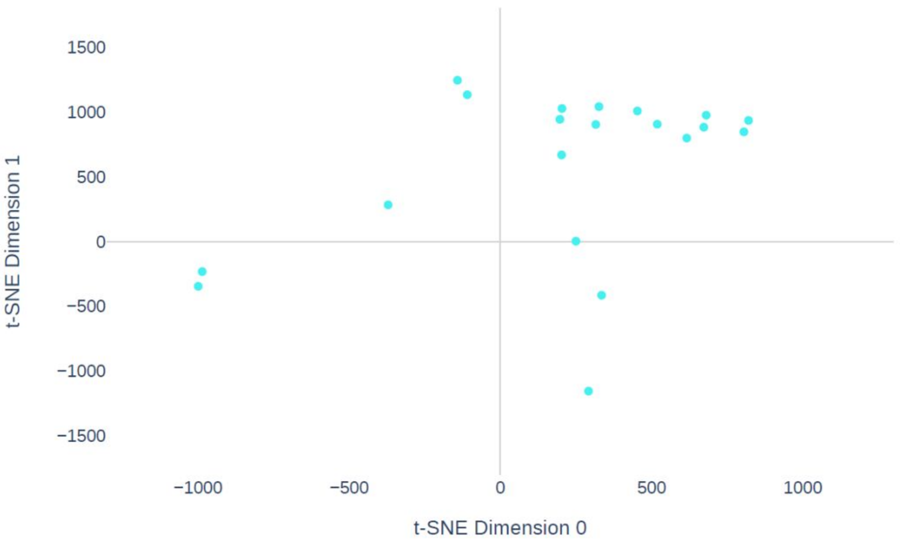

Figure S6: TAS2R4 agonists' space

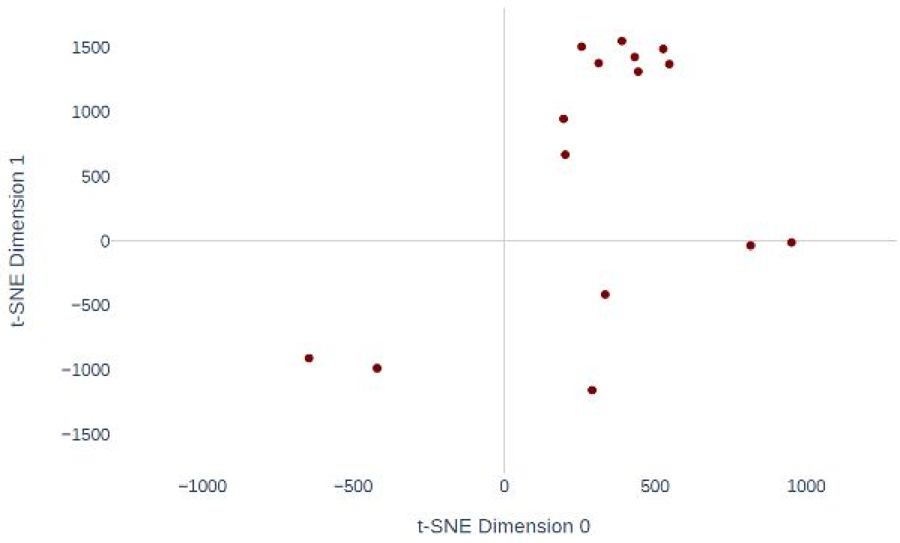

Figure S7: TAS2R10 agonists' space

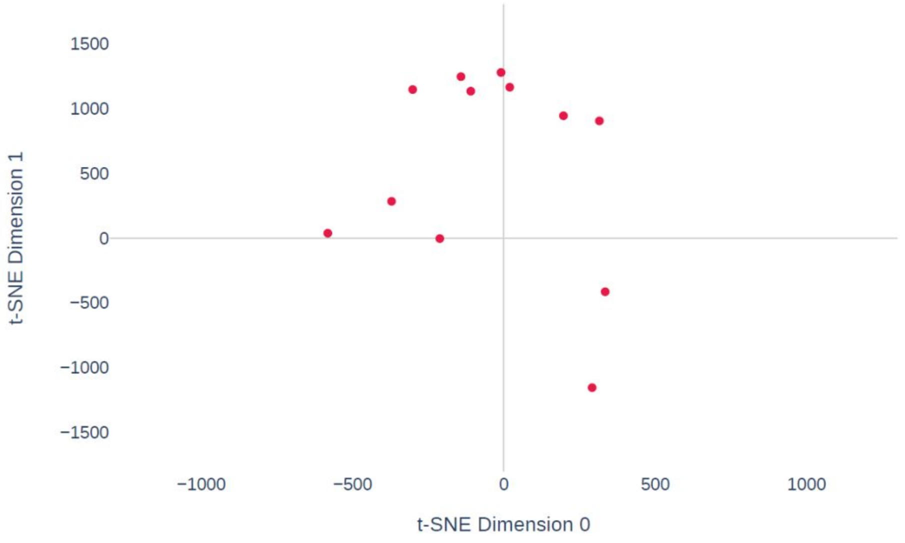

Figure S8: TAS2R1 agonists' space

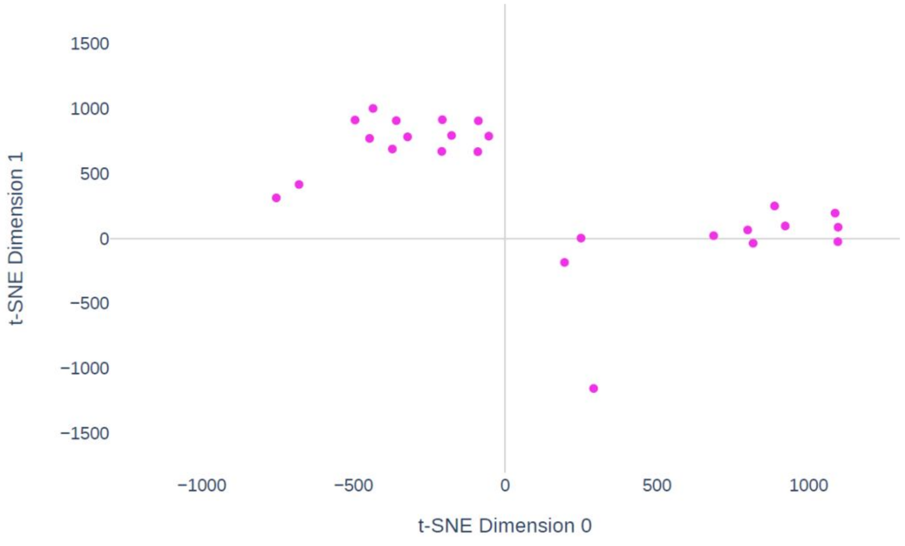

Figure S9: TAS2R38 agonists' space

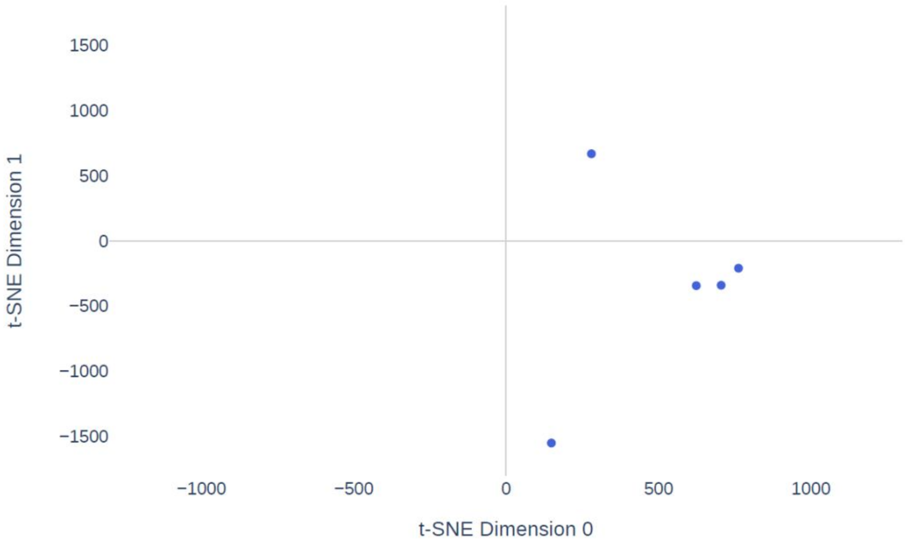

Figure S10: TAS2R30 agonists' space

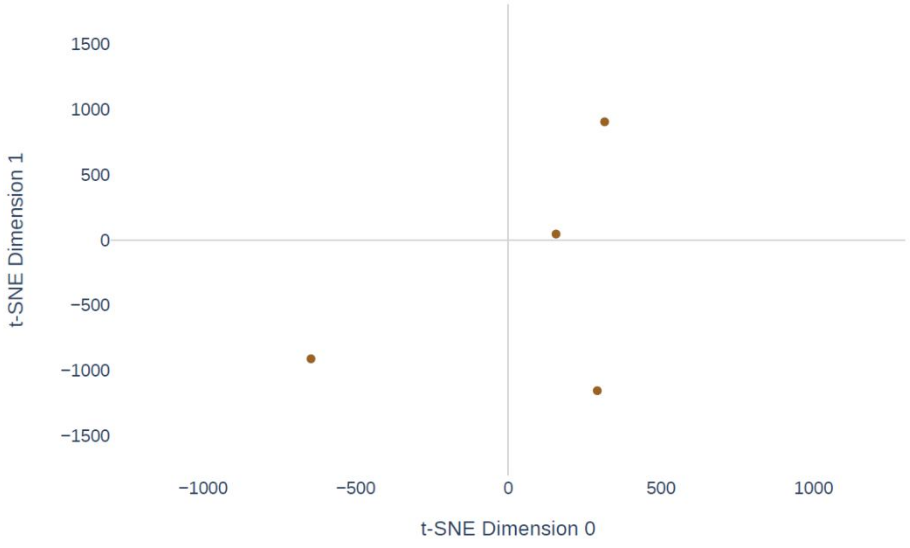

Figure S11: TAS2R40 agonists' space

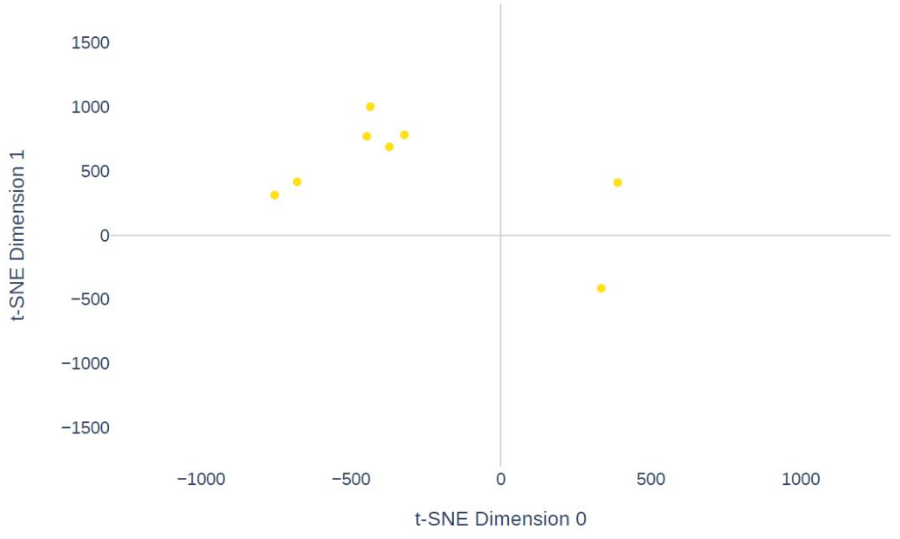

Figure S12: TAS2R43 agonists' space

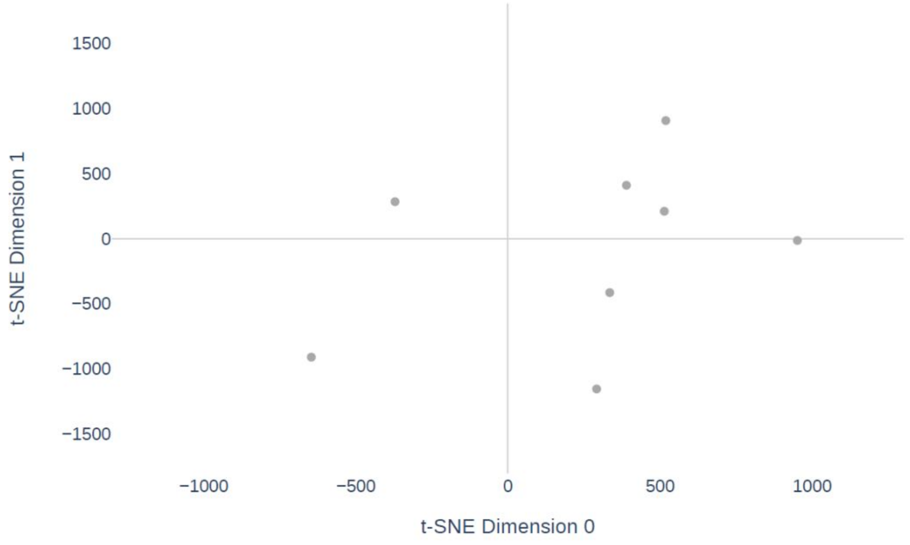

Figure S13: TAS2R7 agonists' space

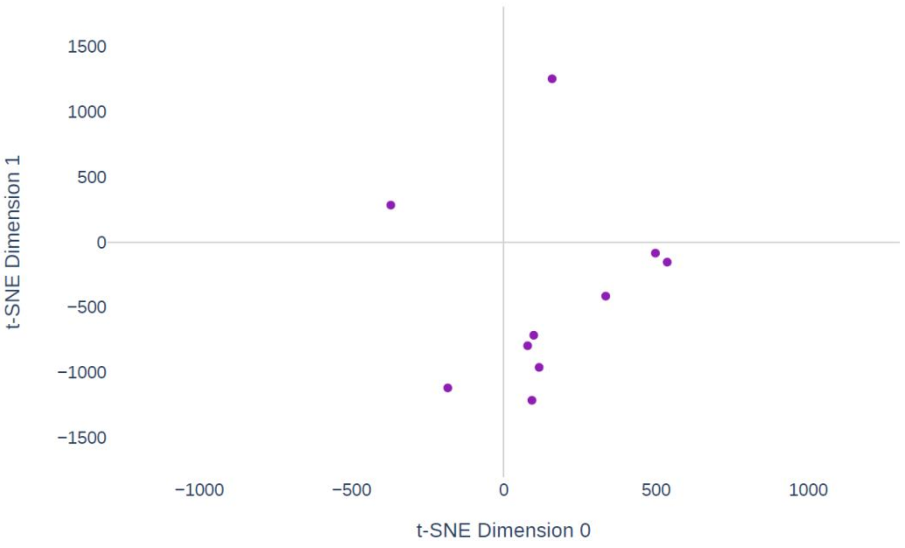

Figure S14: TAS2R50 agonist's space

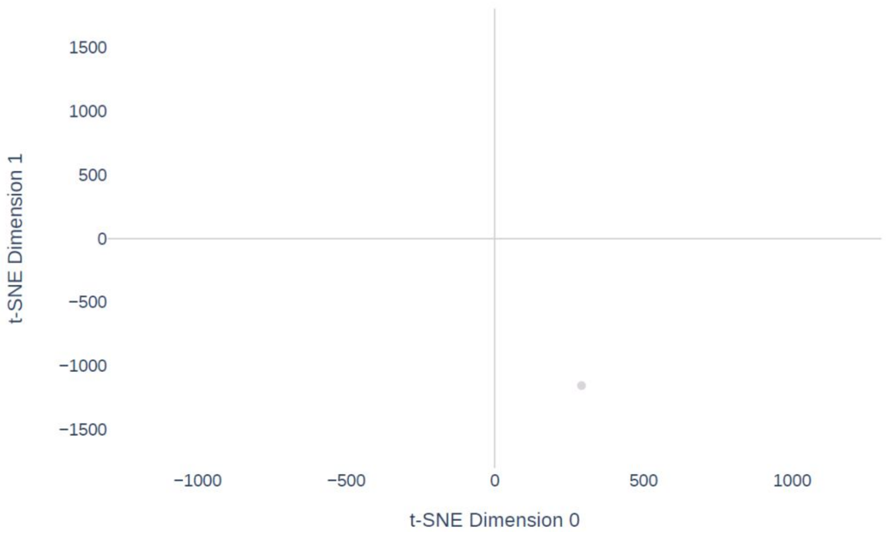

Figure S15: TAS2R31 agonists' space

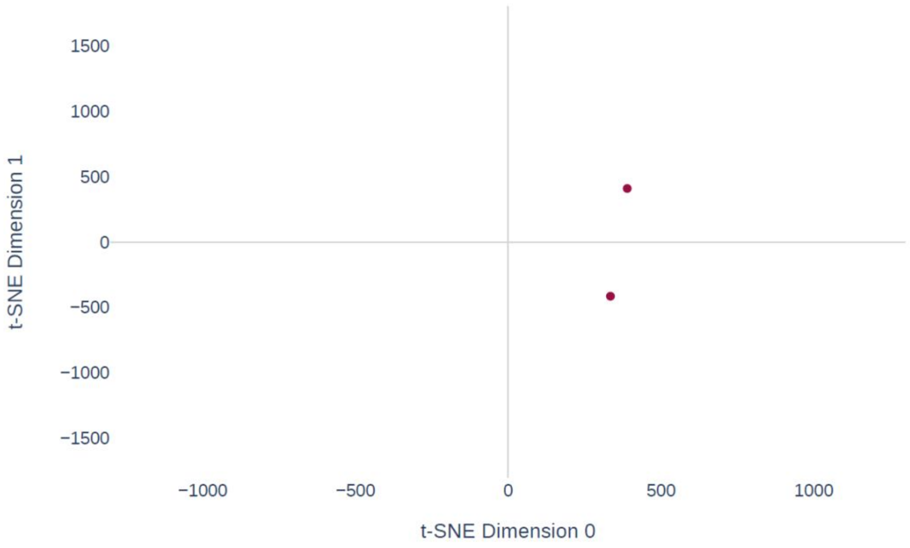

Figure S16: TAS2R31 agonists' space

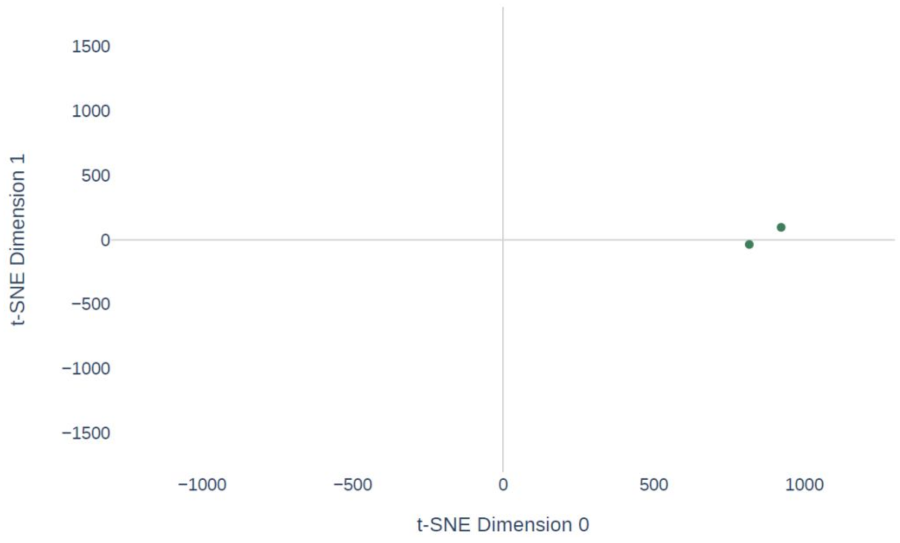

Figure S17: TAS2R20 agonist’s space

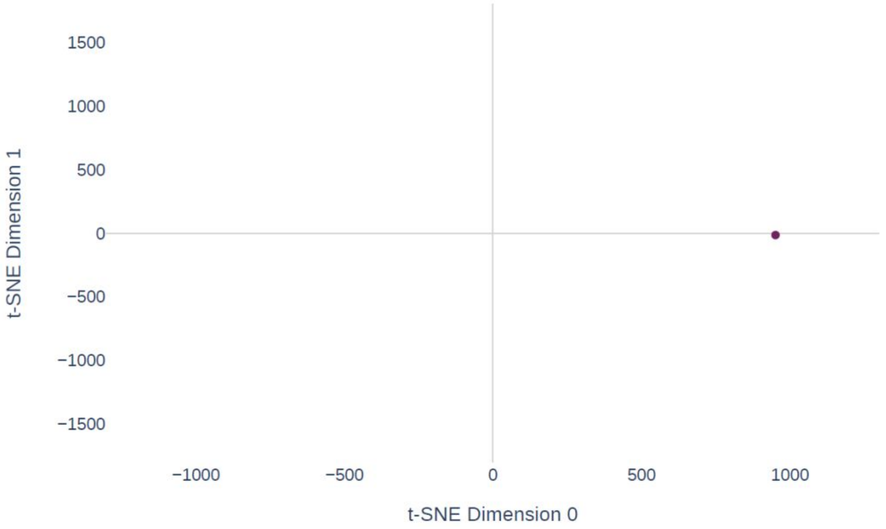

Supplement: Supplementary file 1 — jf1c05057_si_001.pdf [file jf1c05057_si_001.pdf]
